# Pembrolizumab (MK-3475) in combination with lenalidomide and low-dose dexamethasone for relapsed/refractory multiple myeloma (RRMM): KEYNOTE-023

**DOI:** 10.1186/2051-1426-3-S2-P160

**Published:** 2015-11-04

**Authors:** Maria-Victoria Mateos, David Siegel, Jatin J Shah, Donna Reece, David Avigan, Robert Orlowski, Yang Ge, Arun Balakumaran, Patricia Marinello, Jesus San Miguel

**Affiliations:** 1 Complejo Asistencial Universitario de Salamanca/IBSAL, Salamanca, Spain; 2 Hackensack University Medical Center, Hackensack, NJ, USA; 3 The University of Texas MD Anderson Cancer Center, Houston, TX, USA; 4 Princess Margaret Cancer Centre, Toronto, ON, Canada; 5 Beth Israel Deaconess Medical Center, Boston, MA, USA; 6 The University of Texas MD Anderson Cancer Center, Houston, TX, USA; 7 Merck & Co., Inc., Kenilworth, NJ, USA; 8 Universidad de Navarra, Pamplona, Spain

## Background

The immunomodulatory drug (IMiD) lenalidomide has single-agent activity against RRMM, and synergistic effects when combined with dexamethasone. Overexpression of PD-L1 is associated with tumor invasiveness in MM cells and may be a mechanism of immune evasion. Pembrolizumab, a highly selective, humanized IgG4 anti-PD-1 monoclonal antibody designed to block interaction of PD-1 with PD-L1 and PD-L2, may synergize with IMiDs to enhance tumor suppression. KEYNOTE-023 (NCT02036502), an open-label, Phase I, multicenter, nonrandomized, dose-escalation trial will evaluate the safety, tolerability, and efficacy of pembrolizumab in combination with lenalidomide and low-dose dexamethasone in patients with RRMM.

## Methods

Patients ≥18 years with RRMM who have failed ≥2 prior therapies including bortezomib and an IMiD and were refractory to their last line of treatment are eligible. Other key eligibility criteria include measurable disease, ECOG performance status 0/1, and adequate organ function. Key exclusion criteria include history of repeated infections, primary amyloidosis, hyperviscosity, plasma cell leukemia, POEMS syndrome, Waldenström macroglobulinemia, or IgM myeloma; immunosuppressive disorder; prior anti-PD-1/anti-PD-L1 therapy; allogeneic stem cell transplant; or autologous stem cell transplant ≤12 weeks from first infusion. Using a modified 3+3 design followed by toxicity probability interval (TPI) for dose confirmation, cohorts of 3-6 patients per dose level will be enrolled sequentially at escalating doses of pembrolizumab 2, 5, or 10 mg/kg Q2W with low-dose dexamethasone 40 mg and lenalidomide 25 mg until MTD or MAD. Additional patients will receive pembrolizumab in combination with dexamethasone/lenalidomide to confirm the dose based on the TPI algorithm and to evaluate safety and preliminary efficacy. Treatment will continue for 24 months or until complete response, disease progression, or intolerable toxicity. Primary objectives are to establish MTD/MAD and determine safety and tolerability of the combinations. Efficacy end points are stringent complete response, complete response, and very good partial response rate (sCR+CR+VGPR rate); ORR; time to response; duration of response; PFS; OS; and correlation of PD-L1 expression with efficacy. Exploratory objectives are PK and relationship between antitumor activity and candidate biomarkers. Response is assessed using International Myeloma Working Group criteria. AEs are graded per NCI CTCAE v4.0 in all patients receiving ≥1 dose. RP2D will be determined from the dose-response relationship and other tolerability data. Descriptive statistics will summarize efficacy end points for each dose level. Recruitment is ongoing.

## Trial registration

ClinicalTrials.gov identifier NCT02036502.

